# Investigation of Adhesion and Mechanical Properties of Human Glioma Cells by Single Cell Force Spectroscopy and Atomic Force Microscopy

**DOI:** 10.1371/journal.pone.0112582

**Published:** 2014-11-12

**Authors:** Laura Andolfi, Eugenia Bourkoula, Elisa Migliorini, Anita Palma, Anja Pucer, Miran Skrap, Giacinto Scoles, Antonio Paolo Beltrami, Daniela Cesselli, Marco Lazzarino

**Affiliations:** 1 Istituto Officina dei Materiali-National Research Council, Trieste, Italy; 2 Department of Medical and Biological Sciences, University of Udine, Udine, Italy; 3 Département de Chimie Moléculaire, Ingénierie et Interactions Bio Moléculaires, Université Joseph Fourier, Grenoble, France; 4 Cluster in Biomedicine, Trieste, Italy; University of California, Berkeley, United States of America

## Abstract

Active cell migration and invasion is a peculiar feature of glioma that makes this tumor able to rapidly infiltrate into the surrounding brain tissue. In our recent work, we identified a novel class of glioma-associated-stem cells (defined as GASC for high-grade glioma -HG- and Gasc for low-grade glioma -LG-) that, although not tumorigenic, act supporting the biological aggressiveness of glioma-initiating stem cells (defined as GSC for HG and Gsc for LG) favoring also their motility. Migrating cancer cells undergo considerable molecular and cellular changes by remodeling their cytoskeleton and cell interactions with surrounding environment. To get a better understanding about the role of the glioma-associated-stem cells in tumor progression, cell deformability and interactions between glioma-initiating stem cells and glioma-associated-stem cells were investigated. Adhesion of HG/LG-cancer cells on HG/LG-glioma-associated stem cells was studied by time-lapse microscopy, while cell deformability and cell-cell adhesion strengths were quantified by indentation measurements by atomic force microscopy and single cell force spectroscopy. Our results demonstrate that for both HG and LG glioma, cancer-initiating-stem cells are softer than glioma-associated-stem cells, in agreement with their neoplastic features. The adhesion strength of GSC on GASC appears to be significantly lower than that observed for Gsc on Gasc. Whereas, GSC spread and firmly adhere on Gasc with an adhesion strength increased as compared to that obtained on GASC. These findings highlight that the grade of glioma-associated-stem cells plays an important role in modulating cancer cell adhesion, which could affect glioma cell migration, invasion and thus cancer aggressiveness. Moreover this work provides evidence about the importance of investigating cell adhesion and elasticity for new developments in disease diagnostics and therapeutics.

## Introduction

Glioma is the most common primary malignant tumor of the central nervous system and despite recent advances in treatment regimens, the prognosis for affected patients remains still poor [1]. According to WHO classification gliomas can be divided into high-grade gliomas (HGG: anaplastic glioma- grade 3 and glioblastoma - grade 4) and low-grade gliomas (LGG: grade 1 and 2) [1]. Despite optimal treatment, the median survival is 12 to 15 months for patients with glioblastoma and 2 to 5 years for patients with anaplastic glioma [2]. With respect to HGG, LGG grows slowly, but about 70% of grade 2 gliomas evolve to anaplasia, leading to death within 5–10 years [3]–[5]. The highly lethal nature of this tumor partly originates from its invasive characteristics, which allow tumor cells to migrate and infiltrate eloquent areas making impossible the achievement of a radical surgery. Such invasive disease is therefore considered incurable using the treatment modalities presently available [6]. For these reasons, identifying the invasive behavior of glioma may provide diagnostic and prognostic markers, as well as innovative candidate for therapeutic targets. In most carcinomas, it was observed that non-tumor cells (i.e. fibroblast) are present and can favor tumor proliferation, invasion and metastasis [7]. Recently, we have provided evidence of the presence, within human glioma tissues, of a novel class of glioma-associated-stem-cells (defined as GASC for HGG and Gasc for LGG) that grow in adhesion on fibronectin [8]. These cells are devoid of the genetic alterations characterizing glioma tissues, display stem cell features, aberrant growth properties and the ability to modify in vitro the biological features of glioblastoma cells, affecting their growth kinetics, motility and anchorage-independent growth [9]. GASC/Gasc are therefore different from the glioma-initiating-stem cells (defined as GSC for HG and Gsc for LG) that grow in adhesion on laminin and are described as tumor-derived cells able, once transplanted into immunocompromised mice, to give rise to a tumor that is the phenocopy of the patient’s one [10]–[12]. Consequently, we proposed that glioma-associated-stem cells could contribute to the development of a microenvironment that serves as a support for migrating glioma cells [8].

However the mechanism behind the interaction between glioma-initiating cells and glioma-associated-stem cells, likely to play a key role in the tumor progression and invasion, is still not clear. It is known that migrating cancer cells undergo considerable molecular and cellular changes by remodeling cell-cell and cell-matrix adhesion and cytoskeleton organization [13]–[17]. Recent studies have demonstrated that a high cytoskeleton reorganization can affect cell mechanical properties [18]–[22]. Highly motile cancer cells are frequently accompanied by a significant cell softening compared with their healthy counterparts [23], [24] Hence cell adhesion and mechanical features can be considered tightly coupled with the migration process of the cancer cells.

A combined analysis of mechanical and adhesion features of glioma-initiating stem cells with their associated-stem cells can reveal new information about adhesion and migration ability of these cancer cells. Such features have been investigated and quantified by co-culture experiments monitored by fluorescence microscopy and atomic force microscopy (AFM): nanoindentation and single cell force spectroscopy (SCFS). These two modalities enable to perform measurements on single living cells in near-physiological conditions with force resolution down to few pN (i.e. the rupture force of a single hydrogen bond) [24]–[27]. They have been demonstrated to be an effective tool to investigate cell-cell and cell-matrix adhesion [25], [28]–[31], cell stiffness [32], cytoskeleton dynamic [19], [33], specific and non-specific interactions of the cell membrane [34]–[36], which are also involved in tumor cell invasion [37]. Particularly, SCFS allows observing short-term behavior of the cell adhesion process, while standard assays commonly used to study cell adhesion require long time periods (from tens of minutes up to many hours) [34].

In this work we have investigated the mechanical properties of HGG and LGG and the intercellular adhesion of cell sub-populations (GASC, GSC, Gsc and Gasc). Cell-cell adhesion within LGGs and HGGs of isolated sub-populations are analyzed and compared with the inter-populations interactions of GSC with Gasc.

## Materials and Methods

### Cell Culture

A detailed description of the protocol used for isolation and culture of glioma cells from patients is reported in ref [12] and supporting information (File S1). The independent ethic committee of the Azienda Ospedaliero-Universitaria of Udine has approved the research. Informed written consents have been obtained from patients and all clinical investigations have been conducted according to the principles expressed in the Declaration of Helsinki.

### Time-lapse Microscopy to quantify cell adhesion

In order to evaluate the adhesion of glioma-initiating stem cells on the glioma-associated- stem cells, 10^4^ GASC and Gasc cells were seeded in 96-well plates (Black/Clear Imaging Plate, BD-Falcon) for 24 hours and then stained by 5 µM CellTrace CFSE (5(6)-Carboxyfluorescein N-hydroxysuccinimidyl ester, Invitrogen) following manufacturer instructions. GSC and Gsc cells were detached by Tryple (Invitrogen), labelled by 3.25 µM Hoechst 33342 fluorescent solution for 20 min at 37°C and finally plated on the GASC and/or Gasc monolayers (3×10^3^ cells/well). Cells were kept in 5%O_2_/5%CO_2_ incubator at 37°C. GSC and Gsc adhesion to the cell monolayer was evaluated at 30, 60, 90, 120, 180 minutes from cell seeding. Specifically, at each time point selected wells were washed and after the addition of fresh medium images of Hoechst-labeled nuclei as well as phase contrast image and/or CFSE-positive cells were taken by a Leica DMI 6000B microscope connected to a Leica DFC350FX camera (10X objective). Images were then overlaid by Image J in order to evaluate the number of Hoechst positive cells adherent to GASC/Gasc cells as recognized by either phase contrast image or CFSE positivity. In the case of GSC line (n = 2) forming spontaneously aggregates, these latter were quantified as single cells, hypothesizing that only one cell was indeed adherent to the GASC/Gasc cells while the others were adherent to each other. All the time points were evaluated in triplicate for each specified condition. We compared the adhesion of Gsc on Gasc (n = 9) and of GSC on both GASC (n = 7) and Gasc (n = 7).

### AFM experiments

AFM-indentation and SCFS measurements were performed on cells deriving from different patients (n = 3) and cells were generally used at passage 2. SCFS and nanoindentation measurements were performed using a NanoWizard AFM (JPK Instruments, Berlin, Germany) mounted on top of an Axiovert 200 inverted microscope (Carl Zeiss, Jena, Germany). SCFS was performed using a CellHesion module (JPK Instruments, Berlin, Germany) that enables to extend the vertical range of the AFM from 15 µm up to 100 µm to enable complete cell detachment from substrate. All experiments were performed at 37°C using a temperature-controlled BioCell chamber (JPK Instruments, Berlin, Germany). Details about cell adhesion and elasticity measurements are reported in supporting information (File S1).

### Data analysis

The cell adhesion features were obtained by analyzing the retraction curve of force-distance (F-D) curves with the JPK data processing software. Cell mechanical properties were obtained by evaluating the Young’s modulus (E) of the cell. This value was evaluated by analyzing the approaching part of the recorded F-D curves using the JPK DP software. With this option, the software converted the approaching curve into force-indentation curves by subtracting the cantilever bending from the signal height to calculate indentation. Then the fit function described by Hertz-Sneddon model was used (four-sided pyramid as indenter) [38]. To compare the mechanical properties of the different cell sub-populations the measurements were performed at fixed speed (5 µm/sec) and mathematical fits are performed at fixed indentation depth (500 nm): indeed absolute E values were observed to depend significantly on the specific choice of these parameters [16], however, keeping these parameters constant for all experiments, relative E variations were obtained. [16].

### Statistical analysis

The difference in adhesion of GSC on GASC, Gsc on Gasc and GSC on Gasc, respectively, was evaluated by two-way ANOVA followed by Bonferroni post-test. For SCFS data the statistical difference between two groups of data was evaluated by using the non-parametric statistical analysis of the Mann–Whitney test (two-tailed distribution). Histograms of Young’s modulus values were obtained by Origin Pro 8.1. The difference in stiffness between groups of data for cell sub-populations was evaluated by non-parametric Kruskal-Wallis test with Dunn’s multiple comparison test to compare all pairs of column. In all cases, the statistical analysis was performed by GraphPad Prism 5.0. A p value<0.05 was considered statistically significant.

## Results

**Figure 1:**
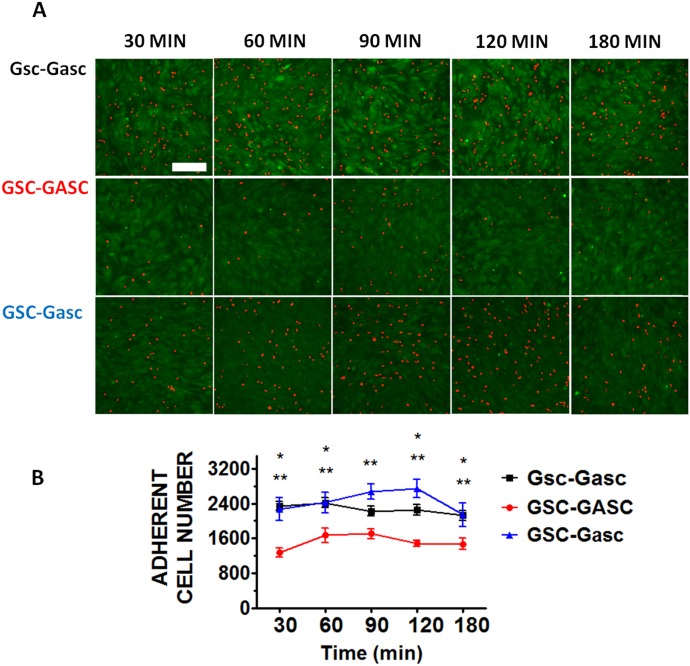
Co-culture of GSC on GASC, Gsc on Gasc and GSC on Gasc. (**A**) Fluorescence images of Hoechst-labeled glioma-initiating cells (red color) on CFSE-labeled glioma associated cells (green color) at different time point (scale bar 200 µm). (**B**) Quantitative analysis of the number of GSC adherent to GASC (red line), Gsc adherent to Gasc (black line) and GSC adherent to Gasc (blue line), respectively. Cell type: p<0.0001, time: p = 0.09. Data are presented as mean ± standard error. *, p<0.05 of GSC-GASC vs Gsc-Gasc; **, p<0.05 of GSC-GASC vs GSC-Gasc.

**Figure 2:**
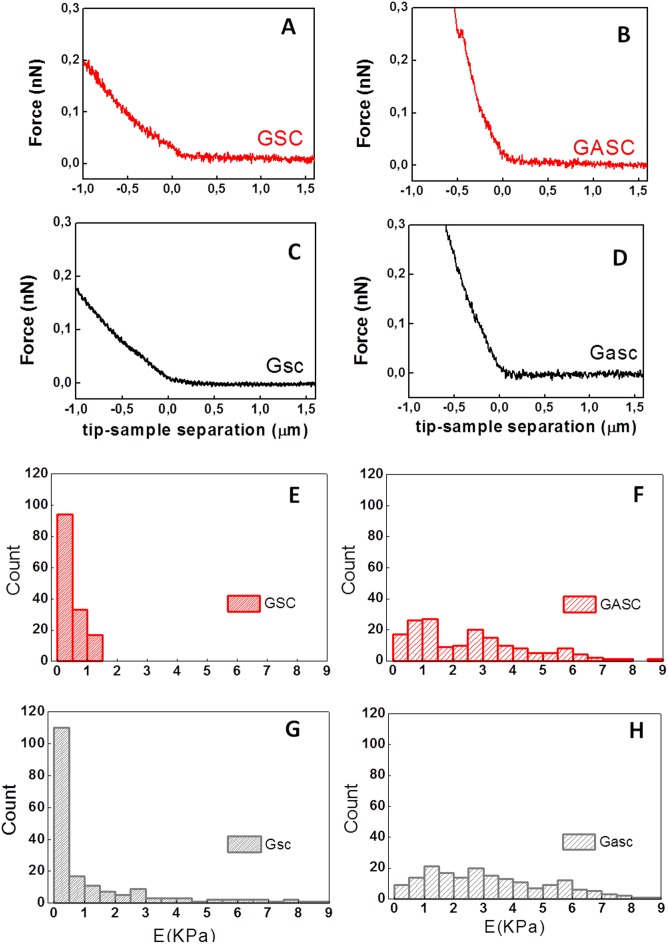
AFM indentation measurements and analysis of each cell subpopulation. (**A–D**) Examples of F-D approaching curves converted into a dependence of load force versus indentations for each cell populations. (**E–H**) Young’s modulus distribution obtained for each cell population. A p value<0.05 is considered statistically significant. GSC and Gsc are significantly softer than GASC and Gasc (p<0.0001); GSC appear also significantly softer than Gsc (p<0.01), while GASC and Gasc do not show significant difference.

**Figure 3:**
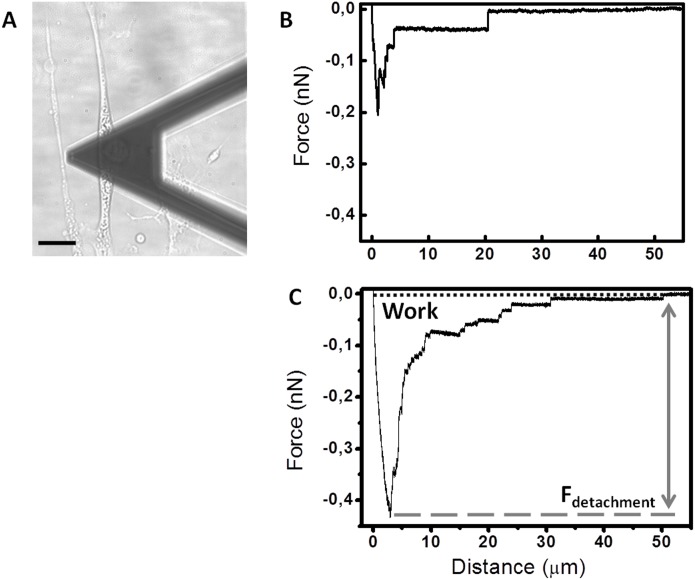
Cell-cell adhesion measurements. (**A**) Differential interference contrast (DIC) optical image of a GSC immobilized on a tipless cantilever brought into contact with a GASC cultured on glass coverslip coated with fibronectin (scale bar 20 µm); representative F-D retraction traces acquired for GSC-GASC (**B**) and Gsc-Gasc (**C**) for 10 sec contact time; features of the curves that enables to quantify adhesion properties are highlited: the maximum force exerted to detach the cell (F_detachment_); the area, included within the retraction curve and the dot line, represents the work done by the cantilever to completely detach the cell from the substrate (work of detachment).

**Figure 4:**
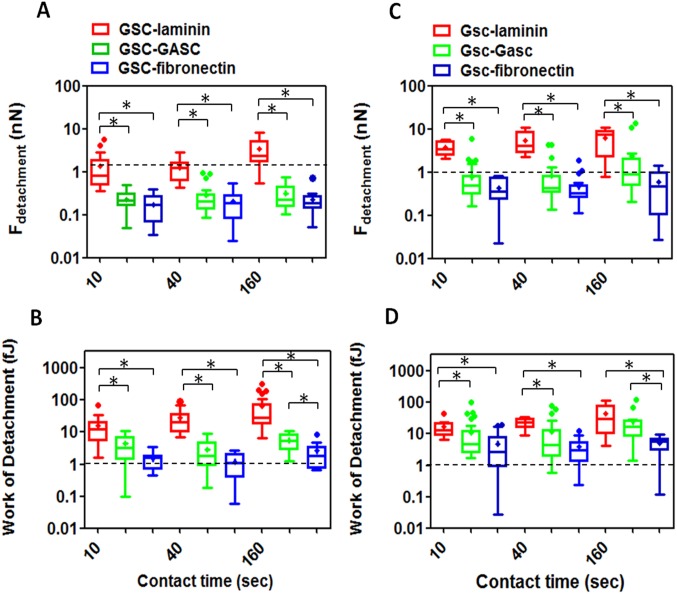
Time dependent analysis of detachment force and work. (**A–B**) Data obtained for GSC-GASC compared with GSC-laminin and GSC-fibronectin and (**C–D**) Gsc-Gasc compared with Gsc-laminin and Gsc-fibronectin; the values inside the box represent the first (25%) and third quartile (75%), while the line within the box represents the median value (50%); the (–) indicate the maximum and minimum observations; while outliers are indicated by (•); the mean value is indicated in the plot as (+); (*) p value<0.05 is considered statistically significant. (**A–B**) Detachment force and work of GSC-laminin are significantly higher than that obtained for GSC-GASC or GSC-fibronectin for each contact time investigated (p<0.0001); No significant differences are obtained between GSC-GASC and GSC-fibronectin, except for work of detachment at 160 sec (p = 0.0097). (**C–D**) The detachment force of Gsc-laminin is higher than that obtained for Gsc-Gasc (p<0.00001 for 10 sec and 40 sec, p = 0.0118 for 160 sec) and Gsc-fibronectin (p<0.00001 for 10 sec and 40 sec, p = 0.0005 for 160 sec). The work of detachment of Gsc-laminin is significantly higher than that obtained for Gsc-Gasc (p = 0.0042) at 10 sec and (p = 0.0006) at 40 sec contact time, while at 160 sec they are no significantly different (p = 0.1167); the work of detachment of Gsc-laminin is higher than that obtained for Gsc-fibronectin for all the contact time investigated (p = 0.0004 for 10 sec, p<0.0001 for 40 sec, p = 0.0006 for 160 sec). No significant differences were obtained between Gsc-Gasc and Gsc-fibronectin, except for work of detachment at 160 sec (p = 0.0004). For a better visualization and comparison of the data, Y scale is reported as Log scale.

**Figure 5:**
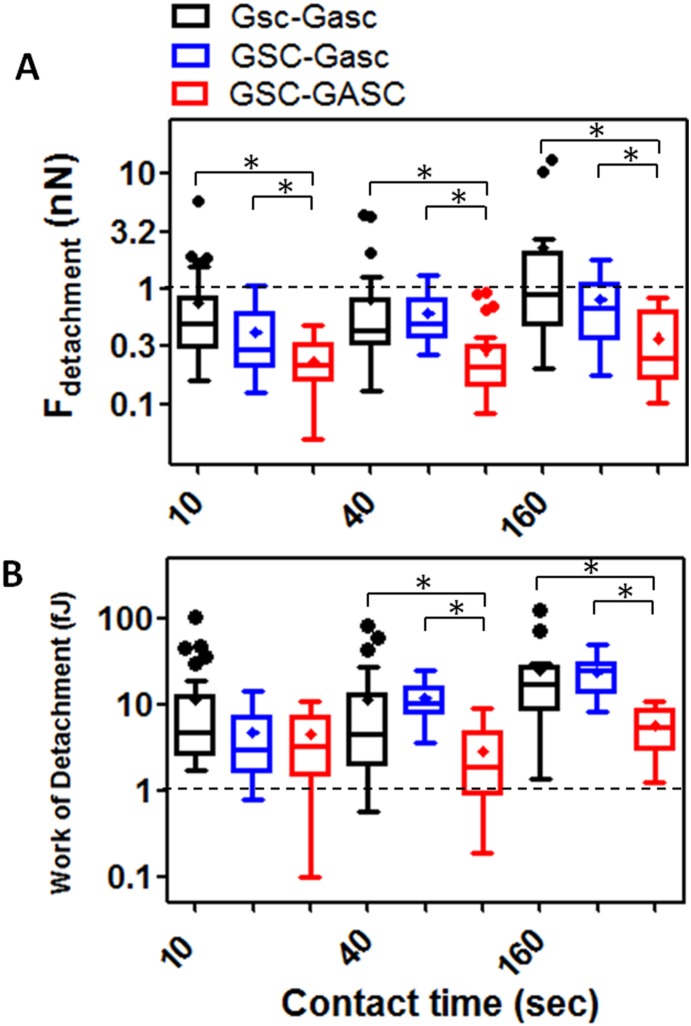
Interpopulation adhesion measurements. Comparison of time dependent detachment force (**A**) and work of detachment (**B**) evaluated for Gsc-Gasc, GSC-Gasc and GSC-GASC; (*) p value<0.05 is considered statistically significant. (**A**) The detachment force of GSC-GASC is higher than that of Gsc-Gasc (p<0.0001 at 10 sec and 40 sec, p = 0.0003 at 160 sec). Detachment force of GSC-Gasc increases significantly as compared to HGG (10 sec p = 0.0267; 40 sec p<0.0001; 160 sec p = 0.0014). (**B**) For work of detachment at 10 sec contact time no significant differences are detected; for higher contact time (40 sec) Gsc-Gasc is significantly higher than GSC-GASC (p = 0.0068) as also the increment of GSC-Gasc as compared to GSC-GASC (p<0.0001); for 160 sec Gsc-Gasc is significantly higher than GSC-GASC (p = 0.0006) as also the increment for GSC-Gasc as compared to GSC-GASC (p<0.0001). For a better visualization and comparison of the data, Y scale is reported as Log scale.

Firstly the cellular adhesion of GSC on GASC; Gsc on Gasc and GSC on Gasc is investigated by co-culturing cells up to 3 hours. Short time lapses have been selected to investigate early stage of adhesion after seeding and for a better comparison with SCFS experiments. As shown in Fig. 1A, the number of Hoechst-labeled GSC and Gsc adherent to the GASC and Gasc are observed at different time points (30, 60, 90, 120 and 180 minutes). Quantitative analysis of the adherent cells demonstrates significant differences depending on the cell type involved in the interaction (Fig. 1B). Specifically, a significantly higher number of Gsc adhere to Gasc when compared to the number of GSC adherent to GASC (Fig. 1B). However, comparing the number of adherent GSC on Gasc with that obtained for GSC on GASC, a significantly superior number of GSC adhere to Gasc, independently from the time point considered (Fig. 1B). In order to evaluate the mechanical properties (deformability) of the different cellular sub-populations, AFM-indentation measurements are performed. In this case an isolated cell is selected out of a cell culture on protein coated coverslip (fibronectin for GASC/Gasc and laminin for GSC/Gsc) using an optical microscope; subsequently the AFM tip (i.e. the indenter) is approached in close proximity of the cell nuclear region and force-distance (F-D) curves are taken. The approaching curves are then converted into force indentation and some examples of those obtained on the four different cell subpopulations are shown in Fig. 2A–D. When a force load is applied to a stiff cell the indentation depth is smaller and the slope of the F-D curve is larger than that observed for a soft cell. By fitting the force-indentation curve with the Hertz-Sneddon model [38], [39], the E value, characterizing the cell stiffness, can be obtained. E values for each cell sub-populations are plotted in the histograms of Fig. 2E–H. Both GSC and Gsc are characterized by a narrow peak at 0.3 kPa, even if Gsc show a broader long tail with E values as high as 8 kPa. On the contrary GASC and Gasc show a wide E distribution with values ranging from 0.3 kPa up to 9 kPa, where the peak at 0.3 kPa is considerably decreased and an average rigidity of 3 kPa- (10 times higher than that observed for cancer cells) can be observed. These data indicate that GASC/Gasc are characterized by a wide mechanical heterogeneity. The statistical analysis demonstrates that GASC/Gasc are significantly stiffer than GSC/Gsc. Moreover, although GSC share with Gsc a high component at low elastic modulus value, the statistical analysis indicates that GSC are softer than Gsc. These results are in agreement with the non-cancer activity and the supporting role of GASC and Gasc as respect to the GSC and Gsc, whose higher deformability is instead in agreement with their neoplastic character [23], [24]. The force interactions between cancer cells and glioma-associated stem cells, both for LGG and HGG are measured and quantified by SCFS. With the help of an optical microscope a single cell (either GSC or Gsc) is picked up by a tipless AFM cantilever functionalized with concanavalin-A. This protein, able to non selectively bind most of the glycoproteins and glycolipids present on the cell membrane, firmly immobilize the glioma cell without influencing its state during measurements. The use of the optical microscope enables to monitor the cell during the measurements, which are interrupted as soon as changes in cell morphology are observed. The cantilever-mounted cell is approached to an isolated cultured cell (either GASC or Gasc) until a 0.5 nN contact force is established. Fig. 3A shows a representative optical image of a GSC immobilized on a functionalized cantilever brought into contact with a GASC cultured on fibronectin coated coverslip. After a predefined contact time, the cantilever-mounted cell is retracted, until the cell is fully detached from the cultured cell. The force with respect to the cantilever position is recorded and F-D curves are obtained. These measurements are performed for increasing contact time (10, 40, 160 sec) and repeated on several cells, by contacting always the body of the cell in correspondence of the nuclear region to minimize the adhesion differences due to the contact with different cellular area. Representative retraction traces of F-D curves resulting from GSC-GASC and Gsc-Gasc interaction are shown in Fig. 3B and C. The analysis of these curves provides quantitative values of the detachment force obtained as the higher adhesion force and the mechanical work done to detach the cell (i.e., detachment energy), which is obtained by integrating the area enclosed by the retraction force curve and the x axis (dot line) (as indicated in Fig. 3C). Same SCFS measurements are performed also for GSC and Gsc brought into contact with either laminin or fibronectin coating to analyze the adhesion strength of cancer cells on both their own culture substrates (laminin) and non-specific substrate (fibronectin). In Fig. 4 the values of detachment force and work obtained at increasing contact time for GSC on GASC (A and B) and Gsc on Gasc (C and D) are plotted in comparison with data obtained for GSC/Gsc on laminin and GSC/Gsc on fibronectin. At each contact time both force and work detachment of GSC on GASC appear to be significantly lower than that observed for GSC on laminin (see Fig. 4A and B) and comparable with those observed for GSC on fibronectin. Indeed significant differences between GSC on GASC and GSC on fibronectin are observed only for work of detachment at 160 sec contact time. Analogous results are obtained for the LGG form (Fig. 4C and D). The high affinity of GSC and Gsc for laminin as respect to that obtained on fibronectin is in agreement with their inability to growth and proliferate on fibronectin [8]. These data suggest that cancer initiating-stem cells have a very low affinity for their supporting cells. It is worth to notice that for the case of Gsc on Gasc, the adhesion properties appear to be widely spread, and at 160 sec contact time no significant differences with the detachment energy of Gsc on laminin could be observed (see Fig. 4D). The latter phenomenon suggests the presence of considerable variability within the cell sub-population, as also observed for wide distribution of the elastic properties of the Gsc (see Fig. 3). In Fig. 5, we compare the detachment force (A) and work (B) obtained for Gsc on Gasc, and GSC on GASC with data obtained for the cross-population measurements GSC on Gasc. In this latter case adhesion experiments are performed by immobilizing a single GSC on the cantilever, which is made to interact with a cultured Gasc at a controlled force for increasing contact time. Here the adhesion strength for GSC on GASC is generally lower than that observed for Gsc on Gasc, except for the work of detachment at low contact time where no differences are found. Instead, the adhesion strength of GSC on Gasc considerably increases respect to GSC on GASC with increasing contact time. At 160 sec contact time the adhesion strength of Gsc on Gasc is 2.32±3.88 nN (mean ± sd) and adhesion energy 24.6±29 fJ, for GSC on GASC is 0.37±0.52 nN and adhesion energy 5.6±3.1 fJ, while for GSC on Gasc they are found to be 0.82±0.52 nN and 23.6±12.9 fJ, respectively. These results confirm that the Gasc are able to enhance and promote the adhesion of highly aggressive GSC. To determine whether a different surface protein pattern could be responsible of the detected differences we perform FACS and immunofluorescence analysis of GASC and Gasc (see File S1). It is found that although GASC and Gasc share a similar surface phenotype, some proteins are differently expressed (see Table S1 in File S1). Specifically, GASC and Gasc differ not only for the expression of stem-cell related markers such as CD133, but also in proteins involved in cell-cell adhesion processes and tumor growth: E-Cadherin (up-regulated in GASC), CD44 and CD105 (up-regulated in Gasc). The different combination of such receptors could be involved in the increased high affinity of GSC for Gasc.

## Discussion

The understanding of the mechanisms activating and promoting the migration of glioma cancer cells into the brain tissue is a fundamental issue to find effective diagnostic tools and alternative treatments to the currently used chemotherapy in order to stop the progress of the disease. Very recently, in the study of cancer migration and invasion particular attention has been devoted to the mechanics of the cancer cells (i.e. adhesion and elastic properties) [40] that beside classical biochemical investigations can improve the knowledge of mechanisms regulating cell migration. The physical interactions of cancer cells with the diverse microenvironments (as extracellular matrix and surrounding cells), encountered during the metastatic process, can have a key role in cancer spreading [23].

In order to get a better understanding of glioma cell interactions with their surrounding environment we have investigated and quantified the mechanical properties and adhesion behavior of glioma-initiating stem cells and non tumorigenic glioma-associated stem cell isolated from HGG and LGG by using time-lapse microscopy, SCFS and nanoindentation AFM. The combination of optical microscopy studies with single molecule techniques, which provide quantitative information about the mechanics of the cell, can allow an extensive investigation of cell-cell interaction. Elastic measurements demonstrate that the cancer cells are softer than glioma-associated stem cells both for HGG and LGG form. The increment in deformability is observed for various cancer cell lines [18], [23], [24], [41] and it is frequently accompanied by alterations of cytoskeleton organization that are known to be also associated with neoplastic transformation [41]. In addition, elastic features appear to be mainly affected by the actin filaments [18], which are highly reorganized in the cytoskeleton of motile cells, being also involved in the formation of migrating cell structures as lamellipodia and filopodia [42], [43]. These findings confirm the supporting non-tumor characteristics of glioma-associated stem cells, in agreement with our *in* *vivo* studies [8], while the higher deformability of the cancer cells may suggest a higher motile character. Moreover, both GASC and Gasc, although showing a broad range of stiffness values, are not mechanically distinguishable, in agreement with the results of genotype and phenotype studies, which found negligible difference in spite of the dramatic differences observed in the progress of the disease [8]. On the contrary, GSC and Gsc show a more pronounced difference: although both have a major component at low E value, the Gsc present a very long tail up to 9 kPa, that might suggest a non-homogenous state of this cell population as compared to GSC. Regarding the cell-cell adhesion, co-culturing experiments demonstrate that HGG and LGG cancer initiating-stem cell and glioma-associated stem cells have different adhesion behavior. The adhesion of GSC on GASC appears to be significantly lower than that observed for Gsc on Gasc. These results demonstrate that the highly aggressive GSC establish weak interactions with their supporting associated-stem cells. Moreover, they point into evidence that HGG shows a cell-cell adhesion profile (both for detachment force and work) more uniform than that observed for the LGG. The cell adhesion variability is a feature already observed in SCFS data and it was demonstrated that this behavior does not depend on cell cycle phase, but originates predominantly from cell to cell variations [44]. However, in our measurements this variability for the LGG could be also associated with the elastic properties of Gsc. The long tail observed in the distribution of Gsc E values suggests that a small percentage of Gsc has lower deformability and as result in the adhesion measurements the cell could contact larger or smaller surface area for same contact force load applied. On the contrary, Gasc are observed to favor the adhesion of GSC increasing the number of adherent cells. Indeed after short time seeding (30 minutes) in co-culture experiment, the number of GSC adherent on Gasc is 63% higher than that observed on the GASC. SCFS data support this behavior even on shorter scale time (few seconds up 3 minutes). When GSC are brought in contact with Gasc, the cell-cell adhesion strength increases, resembling the adhesion behavior observed for the cell-cell interaction of the LG form. These results confirm that Gasc are able to increase the adhesion of highly aggressive GSC. In this case, we can rule out any effect of cellular elasticity on adhesion. Indeed, the difference in elasticity alone between GSC and Gsc subpopulations cannot explain the considerable increment in adhesion observed (see Fig. S1 in File S1). Hence, as suggested by the phenotypic analysis of the surface expression markers, this difference in the adhesion behavior might derive from the different combination of the surface receptors of Gasc and GASC. Our findings underline that intercellular adhesion can play an active role in determining final adhesion behavior that could affect migration ability of cancer cells.

In conclusion, we have shown that glioma-initiating stem cells and glioma-associated stem cells isolated from human glioma tissue have a different deformability, likely related to their neoplastic and non-neoplastic character. We have also demonstrated that highly aggressive cancer cells adhere more strongly on low aggressive associated-stem cells, already at very short time scale (few seconds). The combination of these findings highlight that cell mechanics can play a crucial role in tumor diffusion and that the investigation of these properties could represent an alternative strategy to identify the molecular pathway responsible for tumor invasiveness. Indeed further experiments with specific knock-down of those receptors differently expressed in GASC and Gasc could help in identifying the adhesion molecules favoring cancer cells migration and spreading.

## Supporting Information

File S1 **Detailed description of methodologies and measurements performed: Cell culture.** Flow cytometry and immunofluorescence procedures. AFM indentation measurements. On the role played by cell elasticity on SCFS measurements. SCFS measurements. Table S1 in File S1: Surface immunophenotype of GASC and Gasc. Results are expressed as percentage of cells expressing the assessed marker. Student t-test: significance p<0.05. Figure S1 in File S1: SCFS measurements of GASC/Gasc subpopulation on fibronectin. Comparison of the detachment forces obtained for GASC on fibronectin and Gasc on fibronectin for increasing contact time (For a better visualization and comparison of the data, Y scale is reported as Log scale). No significant differences are observed for all the contact times investigated.
(DOCX)
