# Supplementary material for: Investigation of Adhesion and Mechanical Properties of Human Glioma Cells by Single Cell Force Spectroscopy and Atomic Force Microscopy
Source: PLoS One. 2014 Nov 12;9(11):e112582. doi: 10.1371/journal.pone.0112582 (PMC4229222; doi:10.1371/journal.pone.0112582)
Supplement: File S1 — Detailed description of methodologies and measurements performed: Cell culture. Flow cytometry and immunofluorescence procedures. AFM indentation measurements. On the role played by cell elasticity on SCFS measurements. SCFS measurements. Table S1 in File S1: Surface immunophenotype of GASC and Gasc. Results are expressed as percentage of cells expressing the assessed marker. Student t-test: significance p<0.05. Figure S1 in File S1: SCFS measurements of GASC/Gasc subpopulation on fibronectin. Comparison of the detachment forces obtained for GASC on fibronectin and Gasc on fibronectin for increasing contact time (For a better visualization and comparison of the data, Y scale is reported as Log scale). No significant differences are observed for all the contact times investigated. (DOCX) [file pone.0112582.s001.docx]

**Supporting Information**

**Cell Culture**

LGG (n=3) and HGG (n=3) were mechanically-enzymatically dissociated and cells less than 30µm in diameter cultured in two experimental settings aimed at selectively obtaining either glioma-initiating cells or glioma-associated stem cells. The glioma-associated stem cells were cultured on human fibronectin (Sigma-Aldrich, St. Louis, MO)-coated dishes in an expansion medium composed as follows: 60% low glucose DMEM (Invitrogen, Carlsbad, CA,), 40% MCDB-201, 1mg/mL linoleic acid-BSA, 10^-9^ M dexamethasone, 10^-4^ M ascorbic acid-2 phosphate, 1X insulin-transferrin-sodium selenite (all from Sigma-Aldrich, St. Louis, MO), 2% fetal bovine serum (Stemcell Technologies Inc, Vancovver, BC, Canada), 10 ng/ml human PDGF-BB, 10 ng/ml human EGF (both from Peprotech, Rocky Hill, NJ). Glioma-initiating stem cells were cultured on laminin-coated dishes in a growing medium composed as follows: Neurobasal-A medium (Gibco by Invitrogen, Carlsbad, CA), 2mM L-glutamine (Sigma-Aldrich, St. Louis, MO,), 1X N2 supplement (Gibco by Invitrogen, Carlsbad, CA,), 25mg/ml Insulin, Penicillin-streptomycin, 100mg/ml human apo-trasferrin (Sigma-Aldrich, St. Louis, MO), 1X B-27 supplement (Gibco by Invitrogen, Carlsbad, CA), 20ng/ml h-FGF-basic (Peprotech, Rocky Hill, NJ), 20ng/ml h-EGF (Peprotech, Rocky Hill, NJ). Then cells were cultured in an incubator at 37°C, 5%O_2_/5%CO_2_.

**Flow cytometry and immunofluorescence**

Proliferating cells (n=8 GASC and n=8 Gasc) were detached by Tryple and were incubated with properly conjugated primary antibodies: CD34, CD45, CD49a, CD90, CD73, CD44, HLA-DR, CD146, N-Cadherin (Becton Dickinson, San Jose, CA,), CD133 (Miltenyi Biotec, Gladbach, Germany), E-Cadherin (Santa Cruz Biotechnology, Heidelberg, Germany). Properly conjugated isotype matched antibodies were used as a negative control. The analysis was performed by FACS-Canto (Becton Dickinson, San Jose, CA). Cells cultured in either GASC or GSC medium were fixed in 4% paraformaldehyde for 20 minutes at room temperature (RT), permeabilized for 10 minutes at RT with 0.1% Triton X-100 (Sigma-Aldrich, St. Louis, MO), and incubated over-night at 4°C with: Oct-4 (Abcam, Cambridge., UK, 1:150); Sox-2 (Chemicon, 1:150); and Nanog (Abcam, Cambridge., UK). To detect primary antibodies, A555 dyes labeled secondary antibodies, diluted 1:800, were employed (Molecular Probe, Invitrogen, Carlsbad, CA). Vectashield (Vector Laboratories Inc., Burlingame, CA) added with 0.1 μg/ml DAPI (Sigma-Aldrich, St. Louis, MO) was used as mounting medium. Epifluorescence and phase contrast images were obtained utilizing a live cell imaging dedicated system consisting of a Leica DMI 6000B microscope connected to a Leica DFC350FX camera (Leica Microsystems, Wetzlar, Germany) and equipped with a 63X oil immersion objective (NA: 1.40) or a 40X oil immersion objective (NA: 1.25). Adobe Photoshop software was utilized to compose and overlay the images and to adjust contrast.

**AFM indentation measurements**

For these measurements cells were detached from petri dishes by incubating cells for 1 minute with Tryple (Invitrogen Carlsbad, CA) in the incubator, re-suspended in HBSS (Sigma-Aldrich, St. Louis, MO) and centrifuged for 5 min at 500g. Supernatant was discarded and the cell pellet re-suspended in culture medium. Then, GASC (or Gasc) were seeded on glass coverslip previously coated with fibronectin (10μg/ml for about 30 minutes), while GSC (or Gsc) were seeded on glass coverslip previously coated with laminin (10μg/ml for about 30 minutes) and let both cell subpopulations grow for at least 15 hours before measurements.

For elasticity measurements soft silicon nitrite cantilevers having nominal spring constant of 0.02 N/m (Veeco-Instrument, MLCT-AUHW) were used. Before measurements the spring constant of the cantilevers was calibrated by using the thermal noise method [3]. The mechanical properties of cells were probed by indentation measurements performed by pressing the AFM tip over a single cell. Force-distance (F-D) curves were collected with a force load of 0.5 nN and at a rate of 5μm/sec in closed loop feed-back mode. Measurements were performed always over the nuclear region of the cells, in order to minimize the influence of different contact regions [4]. Over each cell eight-ten F-D curves were acquired, and ten cells for each experiment were measured (for a total number of curves of 150 for GSC; 183 for Gsc; 169 for GASC and 184 for Gasc).

**On the role played by cell elasticity on SCFS measurements**

Performing SCFS on cell populations characterized by different elastic modulus (E) raises some additional methodological questions. Indeed, using the same contact force and contact time for soft and stiff populations may introduce artifacts in the evaluation of adhesion forces. For the same force load, we expect that soft cells have a larger contact area and apply lower pressure on the cell below, while stiff cells have low contact area and exert high pressure on the cell below. These two factors can play an opposite role in establishing focal adhesion points: number of focal adhesion points can increase with contact area, and thus increase adhesion of softer cells, but mechanically activated adhesion points can remain inactive for lower pressure, and thus decrease adhesion of softer cells. In fact, regardless of cell stiffness, when increasing force load, increases both area and pressure and thus adhesion forces. For these reasons in general, when dealing with cell population with different elasticity it is better to keep constant contact area or pressure (i.e. load per unit of area), rather than the overall force load. However, considering the four cell populations investigated in our work we observed that: GASC and Gasc have a very similar distribution of E values, and, concerning the adhesion with fibronectin-coated substrates they are indistinguishable as shown in Fig S1. The statistical analysis indicates that GSC are softer than Gsc, even if Gsc show a broad distribution and share with GSC a high component at low elastic modulus values. Using the median E values (0.34 kPa for Gsc and 0.24 kPa for GSC) the difference in contact area (a) for same force load (F) according to the Hertz contact mechanical model [5] was evaluated. The model describes the contact area between two spheres of radius R_1_ and R_2,_ elastic modulus and Poisson ratio E_1_ E_2_ and ν_1_, ν_2_:

a= ^3^√3/4 F [(1-ν_1_^2^)/E_1_+(1-ν_2_)^2^/E_2_]/ (1/R_1_+1/R_2_)

GSC show an increment in contact area of 20% as respect to Gsc, with an analogous decrease of the pressure. This difference is not sufficient to justify the 600% increase in adhesion force observed between GSC-GASC (0.37 nN) and Gsc-Gasc (2.32 nN) at 160 sec contact time. Moreover, correcting the force load using the median value is far from being rigorous: indeed the cell elasticity can vary from cell to cell and a single GSC cell could be stiffer than a single Gsc cell. Indeed we expect to observe a broader distribution of adhesion forces for a broad distribution of cell elasticity, which is what we observed in the case of Gsc. Therefore, we performed SCFS with same load during the adhesion phase of all the experiments, which, in our opinion is the simplest, clearest and less prone to artifacts approach.

**SCFS measurements**

Adhesion measurements were performed using tipless V-shaped silicon nitride cantilevers having nominal spring constants of 0.32 N/m or 0.08 N/m (NanoWorld, Innovative Technologies). O_2_ plasma treated cantilevers were functionalized with concanavalin-A (Sigma-Aldrich, St. Louis, MO) (incubation 10 μM for 15h at 4°C) and stored in PBS. Before each experiment the cantilever spring constant was calibrated by using the thermal noise method [3]. Cells were detached from petri dishes by incubating cells for 1 minute with Tryple (Invitrogen Carlsbad, CA) in the incubator, re-suspended in HBSS (Sigma-Aldrich, St. Louis, MO) and centrifuged for 5 min at 500g. Supernatant was discarded and the cell pellet re-suspended in culture medium in culture medium and inserted into the BioCell about 20 min after removal from petri dish.

For cell-cell adhesion measurements, Gsc (or GSC) detached from petri dishes, as previously described, was captured by pressing the concanavalin-A functionalized cantilever on a single a cell for 30 sec with a contact force of 0.5 nN against a bovine serum albumin (BSA) layer for which GSC (or GSC) have very low affinity. This protein layer was formed on half glasscoverslip that was inserted into the Biocell just before measurements. Then cell was lifted from the surface and allowed establishing a firm adhesion to the cantilever for about 15 min. Afterwards the cantilever was moved toward a single Gasc (or GASC) previously cultured on fibronectin coated glass coverslip for at least 15h before measurements. Adhesion measurements were performed at a contact force of 0.5 nN for different contact times (10, 40 and 160 sec) in HBSS supplemented with 10 mM HEPES pH 7.0 (Sigma-Aldrich, St. Louis, MO). After each force measurement, the cell was retracted to recover for a period of time slightly higher than the contact time with the surface before adhering to a different spot on the surface. During contact, the piezo height was kept constant using the AFM closed loop feedback mode. The cantilever was withdrawn at constant speed of 5μm/sec over pulling ranges of 70-100 μm to ensure complete detachment of the cell from substrate. For SCFS analysis about 30 force distance curves for each contact time were acquired.

For cell-substrate adhesion measurements, GSC (or Gsc) are immobilized on concanavalin-A cantilever as described above and brought into contact with glass coverslip coated with either laminin or fibronectin and measurements as performed as described above.

**References**

1. Bourne TD, Schiff D (2010) Update on molecular findings, management and outcome in low 587 grade glioma. Nat Rev Neurol 6: 695-701.
2. Stupp R, Tonn JC, Brada M, et al. (2010) High-grade malignant glioma: ESMO Clinical Practice 589 Guidelines for diagnosis, treatment and follow-up. Ann Oncol. 21 Suppl 5:v190- 590 193.
3. Te Riet J, Katan AJ, Rankl C, Stahl SW, Van Buul AM, et al. (2011) Interlaboratory round robin on cantilever calibration for AFM force spectroscopy. Ultramicroscopy 111: 1659–1669.
4. Lekka M, Pogoda K, Gostek J, Klymenko O, Prauzner-Bechcicki S et al. (2012) Cancer cell recognition – Mechanical phenotype. Micron 43: 1259–1266.
5. Johnson K L (1985) Contact Mechanics (Cambridge: Cambridge, University Press) p 452.

**Table S1:**

|  | mean+SD | | | | | | **Gasc *vs* GASC**  (*p* value)* |
| --- | --- | --- | --- | --- | --- | --- | --- |
|  | **Gasc**  N=8 | | | **GASC**  N=8 | | |  |
| CD59 | 99.4 | + | 0.6 | 99.4 | + | 0.4 | 0.976 |
| CD73 | 99.1 | + | 1.4 | 95.8 | + | 4.0 | 0.052 |
| CD13 | 88.3 | + | 32.0 | 99.2 | + | 0.8 | 0.426 |
| **CD44** | 86.4 | + | 22.0 | 52.65 | + | 28.7 | 0.028 |
| **CD105** | 71.0 | + | 40.5 | 22.7 | + | 24.3 | 0.012 |
| CD49A | 74.6 | + | 31.4 | 79.5 | + | 15.3 | 0.700 |
| CD49D | 77.0 | + | 28.6 | 52.9 | + | 28.1 | 0.111 |
| CD29 | 76.1 | + | 25.6 | 64.0 | + | 36.7 | 0.449 |
| CD90 | 82.1 | + | 12.9 | 89.4 | + | 9.0 | 0.208 |
| CD10 | 29.8 | + | 38.6 | 38.6 | + | 25.1 | 0.614 |
| HLA-DR | 0.02 | + | 0.03 | 0.14 | + | 0.16 | 0.280 |
| CD117 | 17.1 | + | 30.2 | 2.7 | + | 3.1 | 0.202 |
| N-CAD | 25.6 | + | 32.5 | 17.5 | + | 20 | 0.592 |
| **E-CAD** | 5.6 | + | 9.7 | 32.2 | + | 32.9 | 0.039 |
| KDR | 2.3 | + | 2.5 | 23.9 | + | 24.4 | 0.040 |
| CD271 | 0.5 | + | 0.7 | 1.0 | + | 0.9 | 0.217 |
| ABCG2 | 1.7 | + | 1.1 | 4.1 | + | 3.6 | 0.144 |
| CD34 | 0.8 | + | 1.7 | 0.6 | + | 0.8 | 0.757 |
| CD66E | 4.3 | + | 6.4 | 28.5 | + | 31.5 | 0.053 |
| CD45 | 0.2 | + | 0.1 | 0.6 | + | 0.4 | 0.145 |
| CXCR4 | 0.1 | + | 0.1 | 0.6 | + | 1.1 | 0.374 |
| CD38 | 0.4 | + | 0.7 | 0.6 | + | 0.8 | 0.777 |
| **CD133** | 0.2 | + | 0.1 | 1.9 | + | 1.8 | 0.047 |

**
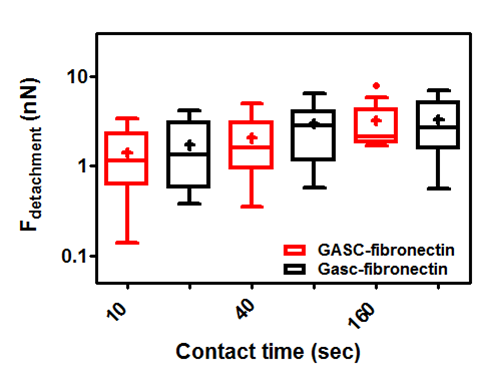
Figure S1:**
